# The Good Life: A Holistic Approach to the Health of the Population

**DOI:** 10.1100/tsw.2006.341

**Published:** 2006-04-14

**Authors:** Said Shahtahmasebi

**Affiliations:** The Good Life Research Centre Trust, Christchurch, New Zealand

**Keywords:** sustainable policy, health care systems, public health

## Abstract

The idea of a holistic approach towards public health planning presented itself through a food-related and trivial curiosity. It is, however, emphasized that food and nutrition are only one aspect of public health. The aim is to reintroduce a holistic approach to achieve sustainable public health with emphasis on the interpretation of the term “holistic”. Holistic decision making is not a new phenomenon and has historical basis. In line with shifts in social norms, decision making has evolved. In particular, various complex models for public health have been proposed to respond to ever-increasing health issues. The advancement in mathematical sciences and technology has led to the quantification of health models. However, mathematical representations pose a major limitation on the holistic approach. Due to its evolutionary nature, human health is dynamically related to social, environmental, and other processes. With the current knowledge, it is difficult to quantify the evolution and feedback effects in holistic models. In this paper, the individual's and public's health is viewed as a dynamic process, but not independent of other dynamic processes (e.g., agriculture, economy, politics) that are all part of a much bigger process. Furthermore, it is argued that it is not merely sufficient to account for all known factors to be holistic. In this paper, the holistic conceptual model is illustrated, using public health as the central issue. The application of the conceptual model is also discussed using two practical examples.

## INTRODUCTION

The idea behind the project arose from wondering about an apparently simple question: How did the outdoor-grown tomato (not perfect in shape, but much more flavorful than ordinary tomatoes) come to be on my plate? Indeed, through the initial stages of developing this project, I realized that the questions we *do not* ask and methods used to research the questions that we *do* ask have changed our relationships with the environment; we have lost touch and harmony with the environment that has been sustaining our lives[1]. The Good Life Project grew from a food context with direct application for health and other social processes. Therefore, it would be reasonable to provide a short background. However, it must be stressed from the outset that food is only one dimension to public health, as is made very clear by the holistic model later in the text.

Over the recent decades, concern has been mounting over a number of food-related issues such as public health, the role of the media, nutritional patterns in children, food production, storage, and delivery. Such issues have been tackled by professionals, politicians, and academics alike, but in isolation, e.g., studies concerned with the diet and health of the socioeconomically disadvantaged[2,3], transport and food[4,5], health education and food[6,7], or government policy on agriculture and food production[8,9]. There have even been studies of modeling human behavior in making choices such as the utility models of food choice[10,11] or brand loyalty models[12]. What such studies have in common is that they all appear to take food for granted and very little attention has been paid to its dynamic and the dynamics of human evolution.

In the past, there have been calls to consider nutrition and diet not just in terms of their relationship to individuals and free choice, but also health and other variables such as socioeconomic, transport, politics, and production[13]. However, such calls are often difficult to realize as workable strategies, as there are a large number of unknown parameters and complex methodologies and designs[14]. For example, in studying cause and effect, we also need to gain insight into the direction of causality and change. The most common approach has been to formulate the unknown into a research question for which research designs may be possible. The literature on food is extensive and covers such diverse issues as health, specific diseases, variations in food components, different methods of agriculture and harvest, etc. Yet we still do not understand the dynamics of food. Do we really have a great deal of food choice? Where does our food and choice of food come from?

One scenario may be to investigate food in terms of consumption, which can be viewed as a human behavior at a number of levels. Individual and household characteristics (e.g., perception, attitude and preferences, politics, socioeconomic), organization and locality (e.g., culture, policies, practices, politics) change over time and are subject to other influences such as politics, food policy, media, and economy. Therefore, consumption changes over time and is subject to a number of influences. These influences are complex processes and cannot be treated as issues or variables, simplified and quantified.

It makes sense to acknowledge the evolutionary nature of food, for the simple fact that it is very closely related to human evolution. This project is about establishing a program of research to encourage the greater involvement of other disciplines to collaborate and share their expertise and knowledge. Discipline is broadly defined and includes identified stakeholders, e.g., academics, policy makers, and various sections of the society. The project is not concerned with starting with a research question to investigate, nor is it about developing one. The idea is that by adopting an inclusive approach, all the other processes related to food (e.g., agricultural, economical, political, social) and actors (stakeholders) will be identified, and common ownership established, thus taking into account the evolution of food. To ensure adequate insight into food, a holistic approach will be essential.

## Limitations of Quantitative Models

A holistic view of human life to address individual and social problems is not a recent phenomenon. It dates back to ancient philosophers and prophets. For example, the Persian philosopher/prophet Zoroast's ideology and training was based on an holistic view of both the physical and mental status; doing good is not enough on its own, and must follow thinking good and being good. Notice that with this holistic model, the three entities are interlinked, emphasizing a "feedback" effect. Similarly, the Ten Commandments, common in most religions and cultures, advocate a holistic approach. Indeed, The Holy Koran has gone beyond the individual, and the rules and training that it offers relate to achieving a spiritually, mentally, and physically healthy society. It is these ideologies that influenced many subsequent philosophers and practitioners who adopted the approach of considering the whole body when treating an illness. The argument behind such an approach has been that physical symptoms could often be the manifestation of underlying unobserved mental (and spiritual) problems and vice versa. Therefore, the interaction between physical, mental, and spiritual elements must be present in the treatment of an illness. There have been many cases utilizing such a relationship with a positive outcome. However, it is usually the unusual cases that attract attention and make history! The following case history would perhaps demonstrate the application of the concept of treating the “whole” person more explicitly.

In a recent case[15], the patient had exhibited psychological symptoms: mainly believing that he possessed mythical and supernatural powers. He insisted on shifting the Rocky Mountains on the outskirts of the city with his bare hands. His family had consulted well-known consultants and specialists and all manner of tests had been conducted and treatments had been prescribed. By the time the last resort was called on, the patient was in a bad shape; he was emaciated and skeletal, and was tied down to the bed for his own safety. The family explained that they had tried everything and that they had heard about the doctor’s healing powers and were hoping for a miracle. After checking the patient’s history, the doctor asked for the patient to be untied. He told the patient that he had come for his help to shift the Rocky Mountains and had heard that he (the patient) was the only one that could help. To the horror of the family, he helped the patient to get organized and they walk towards the front door. The doctor reached for the door handle, but before turning it open, he turned to the patient and said “let’s shake on our new partnership!” The doctor squeezed the patient’s hand so that the patient complained of pain. The doctor persuaded the patient that since his hands are important for this mission, they both should have something to eat and then try again the next day. The patient voluntarily goes back to his room and waits to be fed. In the meantime, the doctor makes up a comprehensive prescription that is mainly dietary. The same pattern of events followed the next day and the next and the next and so on. On what became the final visit, the doctor turned up at the house and examined the patient and said that he was now strong enough to go ahead and help shift the mountains, to which the patient responded “Why?, are you crazy doctor!” In the final analysis, the dietary pattern of the case was held responsible, at least in part, for the psychological symptoms.

As health care systems became more and more complex in order to respond to all the ill-health needs of its population, some practitioners began to equate resources with the rising demand and inequalities in health. The fable of the physician on the riverbank may describe the problem more clearly, retold by McKinlay[16], though attributed by him to Irving Zola:

> “You know”, he said, “sometimes it feels like this. There I am standing by the shore of a swiftly flowing river and I hear the cry of a drowning man. So I jump into the river, put my arms around him, pull him to the shore and apply artificial respiration. Just when he begins to breathe, there is another cry for help. So I jump into the river, reach him, pull him to shore, apply artificial respiration, and then just as he begins to breathe, another cry for help. So back in the river again, reaching, pulling, applying, breathing and then another yell. Again and again, without end, goes the sequence. You know, I am so busy jumping in, pulling them to shore, applying artificial respiration, that I have no time to see who the hell is upstream pushing them all in.”

Thunhirst[17] argued that we have spent too long measuring the flow of the river, the size of the fishing nets, and the rate at which people are brought to shore. Health statisticians and health professionals need to refocus, Thunhirst argues, to investigate the largely unmapped terrain upstream — particularly in solving the problem of differential class experiences of health.

Although, investigating upstream sounds straightforward and the next logical step to take, it is the most difficult. The terrain upstream is made up of and influenced by complex natural and human patterns, which themselves are process outcomes. As Thunhirst points out, the dilemma is one of designing experiments for a long enough period of time and measurements.

Others[18] have argued that health care systems must be viewed as such and proposed a conceptual system where the population’s health is governed by not just health parameters, but by education, economy, transport, agriculture, environment, nutrition, housing, industry, science and technology, medical science, and preventative care[19]. Such a conceptualization would have three layers. At the top layer, you find health factors, natural-biological, and social-economic environments; then the middle layer of working conditions, living conditions, public health, and natural conditions. These two layers are then assumed to influence directly the last layer: social way of life and biological characteristics of the organism.

The difficulty with the IIASA (International Institute for Applied Systems Analysis) model is in how to operationalize it. It was aimed at a universal health care system; far too big and complex even with the level of support and links with other organizations including the World Health Organization. Furthermore, the conceptualization assumed a one-way effect from the top layer through to individual and public health. As demonstrated by IIASA's 1976 conference presentations, the past emphasis had been on mathematical modeling and systems analysis of systems of decision making.

Operationalizing conceptual models is not an easy task. Problems arise when attempting the conventional approach of quantifying and modeling the relationships. How can we quantify relationships that only exist through our conceptualization and we do not have any information on them? Most are processes and process outcomes; and processes are by nature dynamic. Ackoff[20] approached this issue by recognizing that the researcher's concepts may not necessarily match the needs of the subjects being studied. He reported a study in which university resources were provided to the representatives from the ghettos, being researched by a university, to tackle and resolve their own problems. In effect, the subject once being researched was now researching the researchers; a switching of roles. The assumption, quite rightly, was that those living in the ghettos understand the problems better than the nonghetto white researchers. The interesting aspect of this approach was that the "problem", unlike conventional hard approaches, was broadly defined. The problem from the view point of the university was how the new "researchers" would utilize the university resources to solve their own problems.

Therefore, the supply and demand relationship in formulating service provision in a mathematical model is too rigid and often fails to include the human aspects and feedback effects into the equation. Over recent decades, the holistic approach has received much attention, but is still using a systems analysis or systems approach. Studies within the health sector still seem to be "downstream" and more of a system analysis and an evaluation of specific health policies[21,22,23,24,25,26]. Thunhirst[27] argued the need for a new approach that allows participatory research that considers the potential use of operational research for strengthening community participation. The science of problem solving being practiced within a paradigm of problem formulation, optimization, scientization, and depoliticization, and people treated as passive objects, has become inflexible.

Indeed, the working models proposed by various authors[20,27,28,29,30,31] suggest a fundamental move away from the traditional approach of problem identification and formulation into a mathematical model which then could be solved. These approaches rely on methods of identifying the system and its actors, the current state and desired state of the system, and within this framework, adapting methodologies to investigate the system parameters. Such an approach should inform the process of decision making. These essentially nonmathematical approaches to view problems within the environment in which they are conceived are known as soft-systems approaches. It appears that the central emphasis is on the participation of all actors in the system to define and resolve a problem. Without the initial need for quantification and mathematical representation of the problem, it is argued that soft systems allow a greater understanding of the system under study.

However, the applications of soft systems has been mainly within limited environments "downstream" as opposed to "upstream", i.e., with a view to solving "problems". The difference with the conventional mathematical model is then to allow more sources of variation and definitions (e.g., actors, context), but less parameterization. Even with a participatory methodology, problems are identified within the context of organizations and individuals, and groups within organizations/population. In this context, such groups become the problem owners. And even if other social "forces", such as politics, are taken into account, these are considered external in the context of the problem under study. There is ample evidence to support that social influences may not necessarily be external forces; to some extent, health care environment and health policy have been shaped by market forces (e.g. see [32]). And in an attempt to place patients at the center, Lengnick-Hall[33] suggested that patient satisfaction is inadequate to manage complex relationships in delivering health care and proposed a conceptual model for hospital administration where patients play four roles; the patient as supplier, as product, as participant, and as recipient. By defining roles for patients, the model inevitably becomes closed to other influences such as those from the care provider (the hospital). The idea of placing patients at the center is better illustrated by the working model of Cassidy et al.[34]. This holistic model[34] is currently deployed for rehabilitation care development and delivery, which encourages participation without defining roles. Conversely, systems that place resources at the centre have an inherent conflict that will limit their scope in the development of practical and sustainable health care policy. The conflict between health care needs and resource limitations will inevitably allow the narrowing down of the health care to its sub-domain and engage in problem identification relevant to resources. Therefore, the issue becomes the utilization and allocation of the available resources to be responsive to needs, e.g., mental health services, junior doctors' hours, services for the care of the elderly, CHD care, cancer care, etc. Such health care provision is almost entirely based on care provision after the event, i.e., once ill-health occurs. Soft-systems methodologies do advocate a holistic approach to inform decision making, yet they are only holistic within a defined environment. With this definition, the researchers introduce additional and different sets of problems. Olden and Nespoli[35] suggested a model of balancing the competing forces based on the models of Blum[30]. These models assume health as a consequence of divergent, yet predictable, forces that are constantly changing, encapsulated by four forces: environment, heredity, lifestyle, and medical care services. All these are assumed measurable and affecting the next layer; the physical, social, and mental well-being paradigms that compose health. In order for the health services to apply such a modeling approach, the practitioners must, on the one hand, understand and gain insight into the four forces, and on the other, attempt the "measurement" of other nonhealth variables, assuming that there are no inter-relationships between the four forces. Additional problems and complexities that will affect the model outcome arise from the model assumption that these forces are constantly changing. Soft-systems methodologies rarely address issues such as "disease development" or ask the question "Why are we becoming more and more dependent on formal services?" Furthermore, is increased dependence one of the outcomes of attempting to be responsive to the health "needs" of the population? The latter is the feedback effect.

In essence, even with a fundamental shift in our approach to understand and resolve a problem, we are still parameterizing the problem; not in the abstract mathematical fashion, but rather more qualitatively, without attempting to explore the reasons why the problems exist in the first place. Without a good insight into the problem (e.g., disease), the development of possible solutions may become part of the problem in a short period of time. As an example, given the budgetary and resources constraints, and the costs of reorganization, consider the number of health services reorganizations over the last decade alone.

It is all very well to develop conceptual models with diagrams that include all the elements of the system under study. It is quite difficult to ascertain how representative the diagram might be of a dynamic environment such as human nutrition, which involves choice and palate, income, culture, transport and access, nutritional knowledge and information. For example, Wilkinson[36], after a two-stage data dredging, concluded that dietary factors were closely related to mortality rates, and that differentials in mortality due to class may well be determined by differences in diets that are the most important socioeconomic factors. The link between nutrition/diet and health is well documented[37].

Indeed, I ran into difficulties when I attempted to investigate nutrition and nutritional intervention as a health strategy option[14]. The idea was quite simple. We all require nutritional elements in order to survive and function. However, the quantity required may vary from individual to individual, but generally, the consumption of most of the nutritional elements may be bounded by upper and/or lower constraints, such as the recommended daily allowance for fat, sugar, calcium, vitamins, etc. These upper and lower bounds will form a region that could be assumed to be the ideal state (see Fig. 1). On the other hand, the actual nutrient intake may be bounded by choice of food, income, quantity, nutritional values, etc. The actual nutritional bounds will form a different region, which can be assumed dynamic because the bounds are subject to change and variations between and within individuals (see Fig. 1). Such a conceptualization will have implications for policy formation and evaluation. For instance, we could consider developing policies that influence the movement of the dynamic region towards the ideal region to achieve at least a partial overlap.

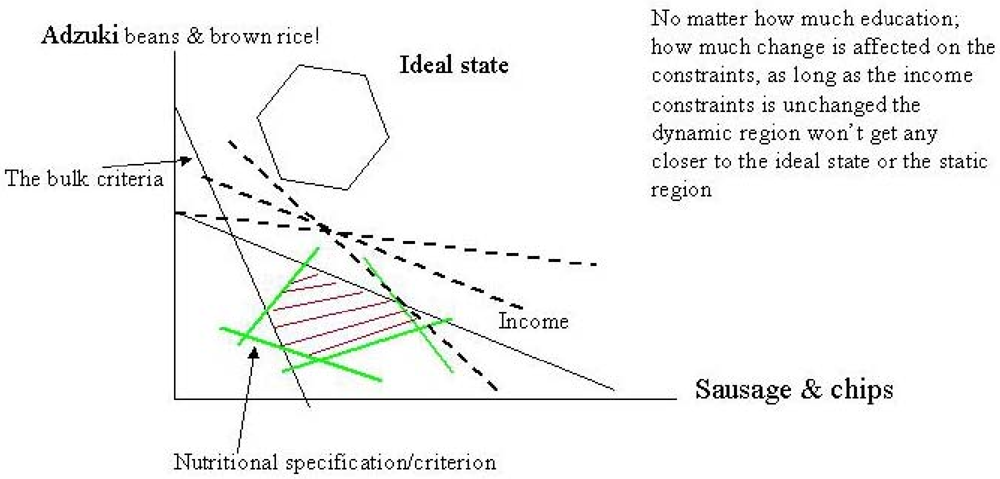

**FIGURE 1.** The model: free choice vs. constraint choice.

Therefore, a good understanding of human behavior is absolutely essential. To gain insight into behavior, we must understand the dynamics of human behavior. In a simple case such as that shown in Fig.1, how can we be sure that a change in circumstances, increase in income, and access to information will lead to the lifting of the constraints, i.e., an overlap between the dynamic region and the ideal state?

The case of the single mother on a low income comes to mind. For those on low incomes, it is not unusual to cut back on food on a regular basis or go without food at least once a week[13]. The single mother in question was a typical case. When she spent the additional money she earned from taking part in an interview to get a hair-do, there was an outcry from the researchers! The expectation was that the respondent would spend the money on food for her children. This example highlights a number of issues: first, it emphasizes the differentials in perceptions and expectations between the subjects and the researchers (as utilized by [20]); second, we cannot be certain about the direction of change; third, we tend to overlook social, mental, and spiritual health and concentrate on physical health when considering the health of the public, which are inter-related.

It can be noted that even though I kept the idea simple and within a two-dimensional framework, the outcome did not stretch beyond a conceptual model. To be holistic, a model of population's health would need to include behavior at three levels: human behavior (e.g., politics, economic, social, health, agriculture), the behavior of the natural environment that we live in (natural resources, climate, land), and the interactions and the feedback effect within and between the two.

## CONCEPTUALIZATION OF A NEW APPROACH

A quick scan of the media's reporting of public policy and a closer examination of governments' policy documents may lead to the perception that there is a randomness about the current policy- and decision-making process. It may be argued that this is an artefact of the democratic model of governance. In countries utilizing the democratic model, governments tend to change over a short period of time. With every general election, ministerial portfolios are expected to change hands. Priorities will change with every election, previously high-priority issues may become low priority during and after the election, and, henceforth, discontinuity in the process of policy development, implementation, and evaluation. On the other hand, the apparent randomness may be due to a utilization of the elective model of decision makers[38] where a decision is made and evidence to support the decision is sought after policy implementation. Furthermore, there is also ample anecdotal evidence to suggest that such an elective modeling approach encourages policies that artificially tackle an outcome rather than tending to the parameters giving rise to the outcome. [The following examples are based on the reports in the media and some government policy documents and are merely illustrative examples. However, it is not within the scope of this paper to carry out a critical analysis of a specific policy.] For example, in the U.K., a government's White Paper introduced and set health outcome targets for the nation[39]. In attempting to achieving targets during the 1990s, the authorities concentrated on the outcome for which targets had been set. Needless to say that by 1997, the new Labour Government suggested that the targets were too specific and broadened the "Health of the Nation" to "Saving Lives: Our Healthier Nation"[40]. The resulting outcome was a missed opportunity to regenerate a relearning process by which the parameters might be identified and better understood, including reasons for having health care systems, hospitals, and various services. Three examples from New Zealand may further help to visualize the difficulties with such a modeling approach.

1. The issue of raising success rates by school pupils has led to a new qualification (NCEA) where the notion of success/failure is minimized and appears to have led to disquiet among teachers and most students (e.g. see http://www.scoop.co.nz/stories/ED0412/S00072.htm).
2. The issue of justice and the pressure on the bulging prison population — Although the option of building more prisons is available, the debate appears to be focused on reducing numbers of prisoners, e.g., taking prisoners out of prison and placing them in the community (e.g., see http://www.nzherald.co.nz/section/story.cfm?c_id=1&objectid=10369195). There is no debate about the dynamic social parameters that may have led to the increased number of prisoners and the authorities' lack of awareness and preparedness.
3. Some time ago, the New Zealand Government introduced an environmental tax, the "flatulent" tax on dairy farmers. The idea was that the gases emitted from the cows were contributing to the greenhouse effect so the farmers must take responsibility through paying an environmental tax. The tax subsequently became the infamous "fart" tax and it was withdrawn after protests (e.g., see http://www.scoop.co.nz/stories/HL0309/S00040.htm.

This model of decision making is also favored by management at all levels; for example, in health and educational establishments where instead of adopting a process by which all actors may be involved to address a common problem, the outcome is manipulated where issues of substance and individuals/groups cannot be distinguished. Therefore, the problem is no more and may be classified as resolved when the individuals/groups have been dealt with. Unlike governments, the management board of institutions may stay in power much longer. The danger is, of course, the establishment of an antisocial culture as discussed elsewhere[41].

The other side of the coin is the lack of attention to the consequences of developing policies and implementing them in this way. The shift in norms, i.e., changes in behavior, perceptions, attitudes, expectations, and general social change is often attributed to time. Although policies are introduced to bring about social change, often such a link is not admitted and the causal relationship to social engineering may be overlooked. All policies will consequence change, be it over a short or long term. Therefore, it is important for policies to be more evidence based and holistic. Furthermore, whether an elective model or evidence based, evaluation and monitoring must be an integral part of the policy to observe the outcome in a multidimension context.

Authors continue to advocate a holistic approach to health care (e.g., see [33,35,42,43,44]) or partnerships between various organizations (e.g., see [32,45]). Most models view health as it is influenced by other phenomena that are often treated as measurable factors or variables such as socioeconomic, politics, natural environment, etc. Such a view of health assumes a "closed" system and even with a participatory approach, inevitably the system becomes bounded very rapidly. In particular, a conflict or clash of interests would be inevitable in a holistic participatory approach, as it would frequently require the participation/collaboration of stakeholders often from "different" backgrounds[44]. Such a conflict may arise from attempts to quantify the model using mathematical formulations. We need a view of health that has other processes (systems) as its neighbors rather than bounds.

## Conceptualization

As illustrated with the model in Fig.2, and the section on quantitative models, the quantification process tends to trivialize and simplify the issue under study. More importantly, such models do not allow for multiple feedback, which is one of the main features of dynamic processes. As mentioned earlier, the project came about as a result of attempting to explore my choice of outdoor-grown tomatoes. They certainly looked different to the ordinary tomatoes and had more flavor. Clearly, previous experience, palate, and preference had influenced this choice, and the extremely short season of the tomatoes may have been influenced by the market, farming parameters, human behavior, social conditioning, etc. Over the years, we have come to accept and indeed expect our food and fruit to have certain characteristics; certain shapes, free from dents and natural bugs, and availability all year round. This, in turn, may have influenced farming and agriculture that, in turn, strengthened the role of storage, transport, technology, and manufacturing. It is plausible that the feedback effect may have been from the latter back to the populations' expectation. On the other hand, how much of our perceptions, expectations, and choices are influenced by health, the environment, and our beliefs? Do media, public health, national food policy, and economics influence our expectations and choices?

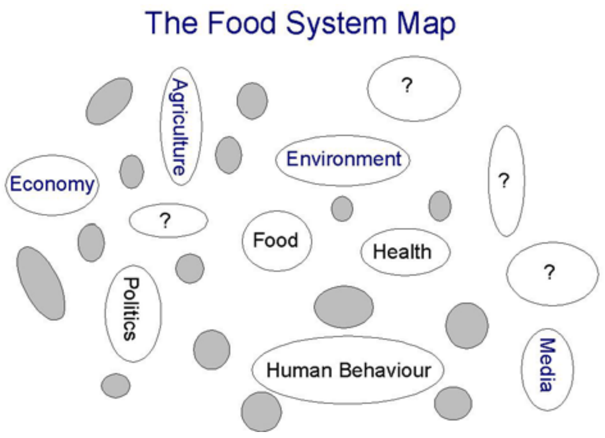

**FIGURE 2.**

It is reasonable to assume behavior into purposeful paradigms, e.g., health, food, media, politics, economics, agriculture, environment, and social. It is also reasonable to assume that these paradigms are processes or dynamic systems. These systems do not just affect "food" in confinement, but are themselves affected by the behavior of food and other systems. For example, food may be affected by agricultural policies and practices, environment, economic policies, and consumer behavior. But, we ought to ask the question: "How much of the environmental effect on food is purely environment and how much of it is through the relationship between agriculture and economic policies and practices?" (see Table 1). Consider the environment as a system with natural components such as climate, to some extent, outside our control. Yet, other components such as land quality, land allocation, water quality and quantity, air quality, pollution, etc. may be affected by agriculture, economy, and politics. At this basic level of visualization, it is possible to notice overlaps between the paradigms.

To explore "food", therefore, the concept described in Fig. 1 is expanded into a multidimensional model in which the ideal state is to accept the current state as a master "system" with all the paradigms as its subsystems. Any changes within one subsystem will impact on that subsystem and all the other subsystems with varying degrees and with a time lag. Similarly, changes in the master system will impact on its subsystems and their inter-relationships.

**TABLE 1**
**Literature-Derived Relationship Between the Subsystems***

| | Advertising | Agriculture | Consumer Behavior | Culture | Economics | Education | Environment | Health | Poverty | Tech |
|---|---|---|---|---|---|---|---|---|---|---|
| **Advertising** | | | | | | | | | | |
| **Agriculture** | | | | | | | | | | |
| **Consumer Behavior** | | | | | | | | | | |
| **Culture/ Demog.** | | | | | | | | | | |
| **Demand** | | | | | | | | | | |
| **Economics** | | | | | | | | | | |
| **Education** | | | | | | | | | | |
| **Environment** | | | | | | | | | | |
| **F safety** | | | | | | | | | | |
| **F security** | | | | | | | | | | |
| **Forestry** | | | | | | | | | | |
| **Globalization** | | | | | | | | | | |
| **Health** | | | | | | | | | | |
| **Income** | | | | | | | | | | |
| **Marketing** | | | | | | | | | | |
| **Nutrition/Diet** | | | | | | | | | | |
| **Organics** | | | | | | | | | | |
| **Politics** | | | | | | | | | | |
| **Population** | | | | | | | | | | |
| **Pollution** | | | | | | | | | | |
| **Poverty/Income** | | | | | | | | | | |
| **Technology** | | | | | | | | | | |
| **General** | | | | | | | | | | |

* The literature search is still continuing and Table 1 is by no means complete. However, it can be seen that the literature already provides some evidence of a relationship between identified subsystems and possibly some of the satellite subsystems. The highlighted cells indicate available evidence of a relationship between the rows and columns. For example, there is evidence of a relationship between economics and advertising/media, agriculture, consumer behavior, health, and poverty. Due to the size of the Table and the volume of the cell references, Table 1 and its associated links and references will be made available for download via The Good Life web-page http://b.1asphost.com/RaDiSol/index%20the%20good%20life.htm.

The conceptualization shown in Fig. 2 assumes a dynamic relationship between the subsystems. No assumptions on the direction of causality are made other than that the relationships may be direct, indirect, or multidirectional. More importantly, the conceptualization assumes a relationship between the subsystems in part through the overlap between them. Consider the simple three-way relationship between politics, the economy, and the environment. A policy decision would directly impact the economy, which may then take some time to affect food. Subsequently, changes in the environment may manifest through the impact on economy and then through the impact on food (not necessarily in that order and not at the same time). Therefore, any environmental policies put in place to counteract the political decisions may appear unsuccessful due to the second wave of impact. By the same token, environmental policies may themselves start a direct impact on economy and food, and subsequently on politics. Such actions and reactions may not be detected so easily in conventional studies. For example, the flatulent tax may be entertaining news, however using Fig. 2, in addition to the social disquiet, the consequences may be better visualized.

The literature provides ample evidence to support a multidimensional relationship between the various aspects of human life. For example, health may be influenced by advertising, agriculture, consumer behavior, and socioeconomic outcomes; by the same token, health may influence these outcomes. These relationships, shown in Table 1, provide the basis of the subsequent conceptualization.

Fig. 2 shows “food” as the central system and all the others are satellite systems or subsystems of food. To maintain the evolutionary aspect, no assumptions are made about the order and relationships between the central system and its satellites. Each satellite is itself a central system having its own satellites. Using the same conceptual approach, it can be seen that the food system may be a satellite system when studying another system such as health, the economy, or the environment. All systems are assumed processes and processes are by nature dynamic.

This step is the foundation of this methodology. The initial investigation involves a major critical literature review (see Table 1) of all aspects of food to map out details of systems conceptualized in Fig. 2. This mapping will be used for all studies within their relevant systems under the Good Life Project to ensure a holistic approach.

## Integration

The integration stage needs to be carried out at several levels, with a single aim to establish that all stakeholders own the “problem” and are therefore part of the solution (as in the mythical patient example):

1. Integration of the literature, past and current studies
2. Integration of diverse disciplines
3. Integration of other actors in the system such as professionals and organizations, including local government, voluntary groups, and other interested individuals

To maintain momentum and the increasing likelihood of success, it was decided to view the study as a small project establishing a research program. The idea is that a handful of researchers will lead their own area of expertise and attempt to link in with “food”. Their responsibility will be to identify and integrate stakeholders into their work.

The integration of nonacademic organizations will be twofold: (1) the inclusion of organizations in the process of project development as either advisor or collaborative colleagues or both, and (2) the inclusion by offer of support.

## Sharing of Ideas and Consultation

Working and researching in isolation is against the ethos of the holistic and evolutionary approach of the Good Life Project. The intentions and mechanism to consult widely are built into the methodology for several main reasons:

1. As part of maintaining the evolutionary nature of the investigation
2. To assist with and ease integration
3. To develop and build cross-discipline bridges and networks
4. To develop and maintain dynamic channels for the flow of information

## RESULTS

### Example 1: Health of the Disadvantaged

In New Zealand, the link between poverty, poor nutrition, and ill health has been clearly identified. According to the New Zealand National Advisory Committee on Health and Disability[46], lower socioeconomic groups have less access to primary health care because what is offered is either not culturally acceptable or it is financially beyond their means. They also have poor nutrition and less knowledge about preventative medical services. This link is clearly reflected in the mortality and morbidity statistics (ibid). In addition to the obvious risk of obesity and heart disease from high-fat intake, individuals/households living in poverty are at risk of conditions such as cancer, diabetes, depression, chronic fatigue syndrome, attention deficit disorder, Parkinson's disease, Alzheimer's disease, multiple sclerosis, and Lou Gehrig's disease because these have all been linked to the lack of vitamins, minerals, and trace elements[47,48,49,50,51]. It has also been noted that following the 1991 benefit cuts and the introduction of market rents for State houses, poverty in New Zealand has increased[52]. With a higher percentage of income being spent on rent and household incomes decreasing, the health-promotion and prevention programs (such as weight loss, antismoking, balanced nutrition, and fitness programs) are out of the reach of those most at risk. These households also have reduced funds to spend on doctor visits, prescription costs, and healthy nutritious food items such as fresh fruit and vegetables (ibid). Policies that allow direct income increase are financially prohibitive and therefore the literature generally concentrate on the characteristics of the groups under study, e.g., provision of food tokens, health education, and health promotion.

#### *The Application of the Conceptual Model*

It is evident that there are a number of processes conceptualized in Fig. 2, i.e., politics, nutrition, food, economy, and health are immediately obvious, while agriculture and media may be acting through food and politics subsystems, see Fig. 3. Applying the approach demonstrated in Fig. 3, the researcher leading this aspect of the project reported some interesting observations. In particular, local and indigenous residents who used to live off the sea and fresh produce from their own back gardens had ceased the practice and perceived it to be more economical to buy take-aways. The ever-changing local social and economical landscape may well be due to an apparent feedback effect from a macro economic policy being manifested locally into fresh fruit and vegetable shops and groceries giving way to fast food and take-away outlets in the deprived areas. The consequences of the resulting change may be, in addition to the adverse effects on health outcomes, the loss of skills that once existed in the community (e.g., acquiring and preparing food from natural sources, cooking). The health of the disadvantaged can no longer be studied in simple terms. These are stakeholders with their own dynamics, thus, adding complexity to the subsystems. All subsystems will have three levels: (1) the macro-level dynamics are due to the government policies and national and regional economy; (2) the micro-level dynamics are due to the individuals/households behavior, local market and demand; and (3) the way (1) and (2) interrelate and affect behavior. For example, how does health promotion affect health and health-related behavior? Does increasing income lead to healthier behavior? The literature seems to avoid such questions. The low-income groups are implicitly assumed to be the problem owners. Help is often through voluntary and charitable organizations (e.g., see [53]). Perhaps by exploring the health of low-income groups as conceptualized in Fig. 3, stakeholders such as the government, local industry, local government, and the neighborhood may be identified also as the problem owners and therefore form part of the solution.

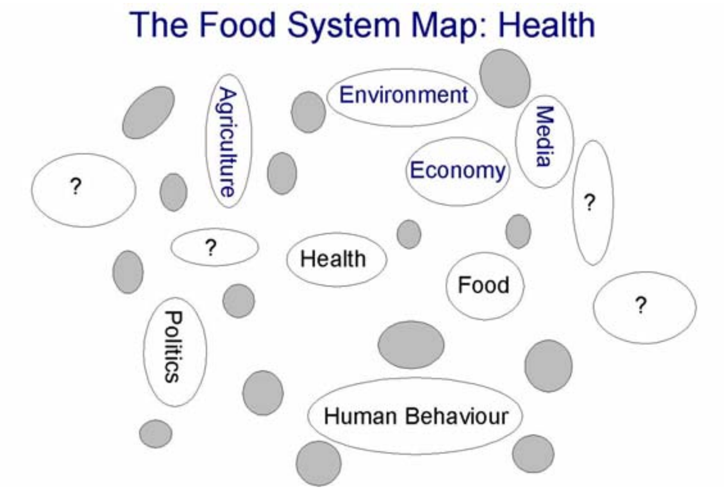

**FIGURE 3**

## Example 2: Cyanobacteria in Canterbury Freshwater Systems

Cyanobacteria (blue-green algae) are a diverse group of photosynthetic bacteria, widely distributed throughout terrestrial, saltwater, and freshwater ecosystems. Within these systems, they fulfill extremely important roles, such as improving soil fertility by fixing atmospheric nitrogen and within aquatic systems providing organic carbon to microbial and animals. Certain cyanobacteria, such as *spirilina* are cultivated as food; others are used in the production of varied pharmaceuticals and in many industrial processes. Some strains produce toxins, including neurotoxins and hepatotoxins[54]. At high exposure, various hepatotoxins cause acute liver damage. Since they are also tumor promoters in mammals, studies in China[55] suggest that long-term exposure to low concentrations may lead to a higher incidence of tumors, such as heptocellular carcinoma.

The impact of cyanobacteria in freshwater systems is being studied more intensively around the world as water quality becomes a pressing issue. In lakes, they form a significant portion of the phytoplankton (suspended in the waters) and periphyton communities (associated with surfaces of substrates such as sediments and rocks). In flowing waters, the greatest biomass is usually in the periphyton. It is well known that eutrophication, caused by agricultural activity and urbanization, stimulates the incidence of nuisance and toxic cyanobacterial blooms[56] that are becoming an increasing common problem worldwide. Codd et al.[57,58] reported that at least 50–75% of phytoplankton blooms were toxic, with frequent livestock death, and less often, human illness and fatalities. Recently, many periphytic cyanobacteria have also been found to be highly toxic and monitoring attempts to reduce their negative impacts consumes considerable resources worldwide[54]. Despite the importance of cyanobacteria, knowledge of their diversity, distribution, and impacts in New Zealand ecosystems is far from complete.

### *The Application of the Conceptual Model*

When applying the conceptual model in Fig. 4 with the environment as the central system, it becomes evident that such a study can no longer be contained in terms of an isolated study of the levels of toxin produced by these cyanobacteria. Again, the subsystems agriculture, food, and health are immediately obvious, while the economy, politics, and media may be operating through agriculture, environment, health, and food. Indeed, Fig. 4 can be conceptualized at a higher level by placing cyanobacteria in place of the environment. The dynamics of the environment (cyanobacteria) as a subsystem and its related subsystems (e.g., water, land, agriculture, human behavior, recreation and food, health, politics, culture,

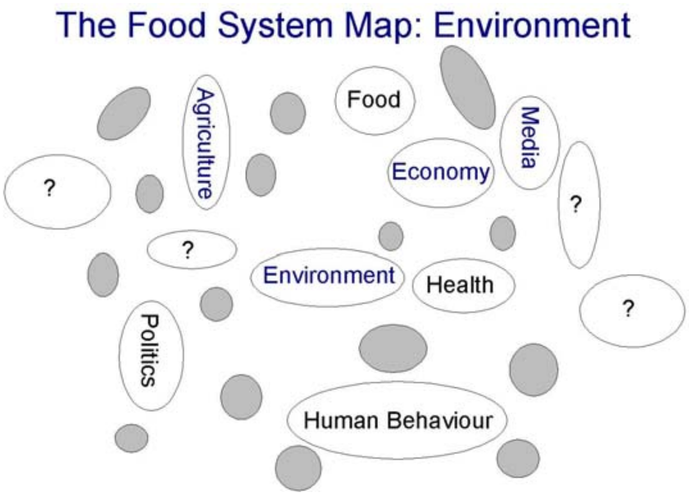

**FIGURE 4**

media, water, land, …) may be visualized when considering a dynamic feedback loop in the relationship between land, water, livestock farms, and food. Further complexities arise from public behavior such as water recreational activities, children swimming in the water, fishing, etc.

## DISCUSSION

Two very different issues (the health of the disadvantaged and cyanobacteria) were used as examples, yet they appear to share the same subsystems. In order to make a positive impact on our living environment, we must abandon the reductionist approach of breaking a dynamic issue into many small problems until it can be viewed as solvable. The notion of problem identification and problem solving based on cross-sectional studies of part of a subsystem will further exacerbate the process. Such an attempt does not lead to sustainable solutions. For example, in the case of the environment, a volunteer spent his time and resources planting native plants along the riverbank with the assumption that the plants would, once established, prevent seepage from the farms into the river. Commendable! When he returned some time later, he was dismayed to see that the plants had been uprooted or destroyed. One is reminded of the black ghetto's research on a university[20] mentioned earlier. In my mind, the apparent success of the research was not entirely due to the switching of roles between the subjects and the researchers, but more to do with all those concerned **wanting** and **willing** to do their bits, both as part of the problem and solution. Therefore, for the riverbank example, it is likely that a small project involving the environment agencies, the farming community, and local neighborhoods to plant native plants along the riverbank would have had more success.

The process of putting this project together — reviewing the literature, attempting to get the project established, and involving others — reinforced human behavior as the main source of the dynamic. Politics. I am reminded of the story of the cat in the court of the lion king: "For reasons of old age, the cat sought permission from the lion king to bring his young son to the court to help him control the mice. On his first day, the young cat's eagerness to impress the king dismayed his father, for he pounced on the mice and eliminated them all in a few moves. Sure, the lion king was impressed at how such a young cat achieved in a few hours what the more experienced cat had not in a lifetime! Father cat spurned the young cat. The young cat was puzzled why he should be spurned – he had done a great service to his aging father and the king; he had rid the court of the mice. He therefore reminded his father the comments made by the lion king. His father said to him that by hastily trying to impress the king and ignoring all the advice and training for the job, he had robbed them of their livelihood; there would not be any mice to annoy the king and the lion would no longer be needing cats! And so the cats were made redundant. Of course with the cats gone, the lion king made some savings on the court's resources, but, subsequently the mice returned and infested the kingdom … " This traditional story captures and expresses the human relationships (politics and priorities at various levels of complexity) and the approach to defining and solving problems very well. We need to adopt an inclusive approach rather than an exclusive one.

It is ironic that universally educational establishments (from schools to universities) and governments are well placed to adopt the application of the conceptual model presented in this paper. The next stage of this project will involve identifying an interested institution as a partner to explore the role of the neighboring systems in terms of change in distance from the central system(s) being studied, i.e., the movement of the neighboring systems when health is replaced by environment as the central system.

Some difficult issues relevant to multidisciplinary collaboration will inevitably arise. It is perhaps appropriate to end this report with the comments from the researchers in the above two examples "A subtle change in attitude can lead to a very different destination," which was in response to the question about "whether to work in isolation or as part of a multidisciplinary team?" It must be emphasized, however, that this project is not about the conventional multidisciplinary practice. As discussed earlier, it is about the sharing of information in a constructive way as to inform the neighboring disciplines.

## ACKNOWLEDGMENTS

The Good Life Research Project was awarded the Montana Special Award for Innovation and Vision, the CPIT Foundation Trust. The original material for Example 1 was provided by Mary Wade and for Example 2 was provided by Dr. Barbara Dolamore. Both researchers were part of the original research team who contributed to an original paper that Barbara Dolamore, together with Matt Morris, presented at the Australian and New Zealand Third Sector Research Conference to at UNITEC, Auckland, New Zealand, 27–29 November 2002. An earlier version of this paper was presented at SIRC 2003 – The 15th Annual Colloquium of the Spatial Information Research Centre, University of Otago, Dunedin, New Zealand, 1–3 December 2003.

## BIOSKETCH

**Said Shahtahmasebi** is currently Principal Consultant, RaDiSol (R & D integrated Solutions) and Research Centre Director, The Good Life Research Centre Trust, Christchurch, New Zealand. He is a research methodologist and statistician. His work experiences are mainly in the U.K. and New Zealand, and cover a number of fields including public health, mental health, nursing, operational research, food and nutrition, and gerontology. His area of interests and expertise are related to longitudinal modeling of health-related behavior. He has founded the Good Life Research Centre Trust to promote holistic research methodology to investigate health, social, and behavioral issues relevant to the community (see http://b.1asphost.com/RaDiSol/). E-mail: said2@slingshot.co.nz
